# Clinical data integration of distributed data sources using Health Level Seven (HL7) v3-RIM mapping

**DOI:** 10.1186/2043-9113-1-32

**Published:** 2011-11-21

**Authors:** Teeradache Viangteeravat, Matthew N Anyanwu, Venkateswara Ra Nagisetty, Emin Kuscu, Mark Eijiro Sakauye, Duojiao Wu

**Affiliations:** 1 Department of Electrical and Computer Engineering, Michigan State University, E. Lansing, MI 48824-1327 USA; 2 Biomedical Informatics, University of Tennessee Health Science Center, Memphis, TN 38163, USA; 3 Vanderbilt University, 2201 West End Ave, Nashville, TN 37235 USA; 4 Biomedical Research Center, Fudan University, Zhongshan Hospital, Shanghai, China, Fenglin Road 180, Shanghai, China

## Abstract

**Background:**

Health information exchange and health information integration has become one of the top priorities for healthcare systems across institutions and hospitals. Most organizations and establishments implement health information exchange and integration in order to support meaningful information retrieval among their disparate healthcare systems. The challenges that prevent efficient health information integration for heterogeneous data sources are the lack of a common standard to support mapping across distributed data sources and the numerous and diverse healthcare domains. Health Level Seven (HL7) is a standards development organization which creates standards, but is itself not the standard. They create the Reference Information Model. RIM is developed by HL7's technical committees. It is a standardized abstract representation of HL7 data across all the domains of health care. In this article, we aim to present a design and a prototype implementation of HL7 v3-RIM mapping for information integration of distributed clinical data sources. The implementation enables the user to retrieve and search information that has been integrated using HL7 v3-RIM technology from disparate health care systems.

**Method and results:**

We designed and developed a prototype implementation of HL7 v3-RIM mapping function to integrate distributed clinical data sources using R-MIM classes from HL7 v3-RIM as a global view along with a collaborative centralized web-based mapping tool to tackle the evolution of both global and local schemas. Our prototype was implemented and integrated with a Clinical Database management Systems CDMS as a plug-in module. We tested the prototype system with some use case scenarios for distributed clinical data sources across several legacy CDMS. The results have been effective in improving information delivery, completing tasks that would have been otherwise difficult to accomplish, and reducing the time required to finish tasks which are used in collaborative information retrieval and sharing with other systems.

**Conclusions:**

We created a prototype implementation of HL7 v3-RIM mapping for information integration between distributed clinical data sources to promote collaborative healthcare and translational research. The prototype has effectively and efficiently ensured the accuracy of the information and knowledge extractions for systems that have been integrated

## Background

Health information integration (HII) is one of the crucial challenges for healthcare systems to integrate heterogeneous data sources into a standard format. This process allows researchers to extract information and knowledge from the integrated systems effectively and efficiently using information retrieval (IR) methods, such as semantic similarity mapping [1]. Many practices realize that performing integration tasks in legacy health information systems is a cumbersome one due to the restrictions in semantic interoperability requirements. The lack of standards is a barrier for the electronic health record (EHR) implementation and integrated delivery systems which support health information exchange (HIE) in disparate health information management systems (HIMS) [2]. In order to integrate the discussed standards, a unified methodology is desired, one that applies different technique, informational and computational, and use standardized terminologies such as the following: the International Classification of Diseases, Ninth and Tenth Revisions (ICD-9 and ICD-10), Current Procedural Terminology, 4th Edition (CPT-4) and Systematized Nomenclature of Medicine-Clinical Terms (SNOMED CT). These are important requirements for all information models that represent the health information in a standard way across all the domains of healthcare.

With the use of well defined and widely accepted standards, information can be integrated and shared within and across healthcare domains enabling HIE to be implemented. With the vision of an integrated system, there should be only one integrated schema (global view) that combines all distributed data sources across several disparate systems and allows the integrated system to seamlessly and efficiently extract relevant information and evaluate the effectiveness of HIE. Various information integration techniques have been proposed in the past, for example schema integration [3,4], data warehousing [5], federated databases [6], distributed and adaptive distributed query processing systems [7,8].

Srikrishnan et. al. proposed an integrated system from distributed databases using the hyper-graph data model which represents database schema in a low-level data model [9]. Several database integration projects were developed in global-as-view (GAV) technique, in which the global schema was constructed as a view over the local schemas [10]. The drawback of GAV is the evolution of local schemas when existing local schemas are changed and all the predefined information retrieval processes have to be reconstructed to adapt to the new design. The need for greater flexibility in changes in local schemas was addressed by local-as-view (LAV) technique [11]. However, the drawbacks are more complex in query processing time in LAV than in GAV [9]. Mazzarisi et. al. developed the integrated data system for the electrophysiology laboratory (EPH-Lab) using HL7 clinical document architecture (CDA) [12]. Deigo et. al., proposed a point-to-point interface architecture and the message server integration techniques as the mediator integration model using HL7-based legacy systems integration [13].

Existing legacy clinical data management System (CDMS) refers the system that is in used before the introduction of HL7 based system (v2 or v3), while all references to "HL7 legacy systems" refer to HL7 v2 implementations since v2 is the older of the two technologies. A comparison of HL7 v2 and v3 messages reveals that they are incompatible with each other, as their message formats are completely different. v2 messages use delimiters ("|" symbol) to separate values, whereas v3 messages use Extensible Markup Language (XML) data objects for values. Also, HL7 v2.3.1 and later versions support XML encoding of messages, but the HL7 v3 XML specification is built around vocabularies and data types appropriately extracted from RIM [14,15]. v3 was designed with stronger standardization in mind, so v3 messages are much more consistent with each other than v2 [15]. The v2 specification is commonly represented using ASCII (American Standard Code for Information Interchange) format. The XML tags are based on natural language; thus it can be both read by humans and processed by machines. This attribute enables a higher level of semantic and syntactic interoperability from one system to another [16]. Another limitation of v2 is that the standard messages have a large number of optional segments and fields, thus it cannot be implemented easily. These preclude rigorous conformance testing. Additionally, the v2 standard does not lend itself to implementation in alternate communication protocols. Some of the benefits of HL7 v2 messages include, but not limited to, ease of implementation, backward compatibility with other HL7 v2 versions, the capability of being implemented in modules, the provision of an application program interface (API) for interfacing with legacy systems by providing 80 percent of the interface frame work [13-16].

Scientists and researchers have been using one technique or the other in order to integrate their CDMS legacy systems and HL7 v2 systems with the HL7 v3 system. In an effort to build a unified format for a National Information System in Turkey, The Ministry of Health implemented the Clinical Document Architecture Release Two (CDA R2) standard and a client application compatible with HL7 v3 for information interchange [17]. Yang et. al. presented the design of the HL7 RIM based sharing components for clinical information systems in Taipei City Hospital [18]. Sui-hui et. al. developed the HL7 v3 gateway using Web Services aimed at solving the bottleneck of the HL7 v2.x standard for data transfer between two medical information systems [19]. Paterson et. al. proposed a boundary objects approach by designing an HL7 template for data entry against information codification such as HL7 vocabulary, HL7 external vocabularies and controlled vocabularies in order to improve the quality of the data in the discharge summary [20].

In this article, we developed a prototype to integrate distributed clinical data sources using R-MIM classes from HL7 v3-RIM as a global view along with a collaborative centralized web-based mapping tool to tackle the evolution of both global and local schemas. Our prototype was implemented and integrated with a clinical data management system as a Plug-in module using a CDMS. A clinical data management system (e.g Slim-Prim [21,22]) is used in administering and managing patient medical records and making the records available to health-care providers so that they can be used in their research and translational health care practice. We have tested the prototype system with some use case scenarios for distributed clinical data sources across several legacy clinical database management systems (CDMS) and database management systems (DBMS) at the University of Tennessee Health Science Center (UTHSC). These disparate systems were built on different underlying database technologies such as Oracle, MySQL and MS Access. All the database management systems (Oracle, MySQL, and MS Access) used are relational database model, as this ensures a one-one, one-many or many-many relation between a patient's administrative and clinical health information data items. The results have been effective in improving information delivery, completing tasks that would have been otherwise difficult to finish, and reducing the time required to accomplish tasks which are used in collaborative information retrieval and sharing with other systems. One of the challenges implementing HL7 v3 is creating automatic semantic interoperability for existing legacy CDMS and also with HL7 v2 format.

The HL7 technical committees developed the HL7 v3 message structure that is based on the reference information model (RIM). The objective of this model is to tackle the bottleneck involved in the information interchange among health information systems. The RIM is the data source, with a coherent and shared information model that is necessary for the data content of all the HL7 v3 messages. The RIM is an abstract model-driven development methodology based on a unified modelling language (UML) and the root of all information models that represent the HL7 data in a standard way across all the domains of healthcare system. It is also a complete HL7 v3 reference model that includes all the object attributes and properties and state transition diagrams that specify the life cycles of all class objects [23]. For specific domains, the Domain-Message Information Model (D-MIM) represents a refined subset of the RIM that is used to drive domain-specific information models such as "Administrative Management Domain -Accounting and Billing" and "Health and Clinical Management Domain-Clinical Document Architecture Medical Record." A D-MIM is composed of a set of class clones, attributes, state-machines and relationships in R-MIM that are essential for constructing HL7 v3 messages for a particular domain in a specific area of interest in healthcare.

RIM core classes contain the following subject areas: Acts, Entities and Roles. The Acts subject area contains the following classes: Account, Act, ActRelationship, ControlAct, Device Task, DiagnosticImage, Diet, FinancialContract, FinancialTransaction, InvoiceElement, ManagedParticipation, Observation, Participation, PatientEncounter, Procedure, PublicHealthCase, SubstanceAdministration, Supply and WorkingList. All the classes in the Acts subject area relates to all the events and actions in the health care services [24]. The Entities subject area consists of the following classes: Container, Device, Entity, LanguageCommunication, LivingSubject, ManufacturedMaterial, Material, NonPersonLivingSubject, Organization, Person and Place. All the classes in the Entities subject area involve all the stake holders in the health care services. Role subject classes are Access, Employee, LicensedEntity, Patient, Role and RoleLink, and they relate to roles the participants play in health care services.

Other subclasses can be derived or cloned from the core classes (e.g., observation and procedure subclasses derived from the class act). The clone classes can be viewed as a direct or conceptual specialization of the core class. There are invariably thousands of clone classes with the core or specific domain classes of RIM [24].

Once the domain is specified, a Refined-Message Information Model (R-MIM) is used to express the content for a set of messages with incumbent annotations and elaborations that are message specific. To exchange information between systems, the Hierarchical Message Descriptions (HMDs) represents the message structures or message types that are used to express R-MIM abstract message structures in an organized way, which can be communicated between systems with disparate underlying technologies. One of the most important things the HMD does is to specify the serialization of the two dimensional R-MIM into a one dimensional data stream. It also involves the Implementation Technology Specification (ITS) through Extensible Markup Language (XML) and Unified Modeling Language (UML) [25,26]. Furthermore, since HL7 v3 specification is based on RIM classes and v3 message uses XML that includes both data and metadata in a unified format, the data (XML representation) can be correctly processed at its destination point irrespective of the platform or technology that may evolve in the future. This is to enable a higher level of semantic consistency and interoperability for the interchange of clinical data, biomedical data, and other data from one system to another.

The HL7 data types and structures are defined by XML ITS. It follows the extendable markup language protocols, while the structures represent the constructs defined by HMDs. Thus for every HL7 message type it is necessary to have an HMD and XML Schema Definition (XSD) to express a set of rules to which XML document structure must follow in order to be considered valid according to the schema specifications. Thus the XSDs contain all required information that is essential for constructing a complete HL7 v3 message.

An understanding of the artifacts for domain-specific models is essential for understanding the HL7 v3 specification. For every domain, the artifacts are organized in the same structure that is submitted by the HL7 technical committee during the specification development process. For instance, an application role submitted by the Patient Administration under Administrative Management Domain will have a unique artifact identifier like PRPA_ IN101001UV 01 where PR = Practice (Subsection), PA = Patient Administration (Domain), IN = Interaction (Artifact type), 101001 = 6 digit non-meaningful number assigned by the Technical Committee to ensure uniqueness, UV = Realm (the only current value is UV for universal), and 01 = Current version number. The root element uniquely identifies the message's interaction identifier, which identifies the message type, the trigger event, and the receiver responsibilities. In this example, the interaction between two systems is defined by the interaction code (IN). The IN shown in the actual message would be composed of the Trigger Event, the Message Type, the Transmission Wrapper, the Control Act Wrapper, the Sender, and the Receiver. The Trigger Event and Control Act Wrapper represent another wrapper around the actual message, which explains information about the date and time the trigger event occurred as well as the responsible parties for the trigger. The development of RIM opens the door to significant movement from HIE standards through messaging to an integrated healthcare systems architectural paradigm. RIM represents all the attributes and data elements that are needed for HL7 message communication and data exchange [23,25].

Compared with other implementations of Health Information exchange [9,10,12,13], our prototype implementation of HL7 v3 ensures the mapping for information integration between distributed clinical data sources to promote collaborative healthcare and translational research. This mapping is triggered in real-time to ensure that the right information is received at the right time. Our approach has effectively and efficiently ensured the correctness of the information and knowledge extractions for systems that have been integrated. Our prototype integrates distributed clinical data sources using R-MIM classes from HL7 v3-RIM as a global view, along with a collaborative centralized web-based mapping tool to tackle the evolution of both global and local schemas.

## Methods and results

### Clinical data integration workflow

**Figure 1:**
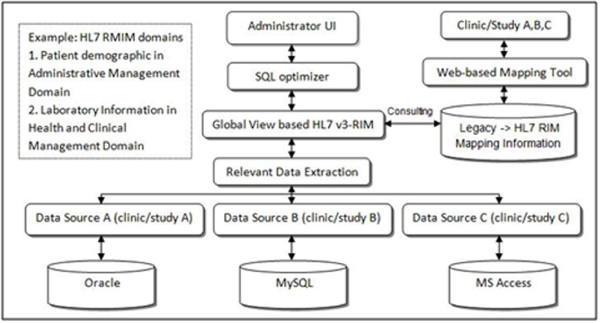
**Clinical data integration workflow using HL7 v3-RIM mapping**. Clinical data integration workflow using HL7 v3-RIM mapping function, showing the developed integrated prototype with several key functions.

The integrated system we developed consists of several key functions, as shown in Figure 1. We selected R-MIM classes that represent patient demographics in the administrative management domain and public health reporting in the health and clinical management domain as our pioneered scenarios. Accomplishing this integration workflow is very important as it will enable us to apply it to other R-MIM classes and expand it through a broader integration across a wider range of information systems.

**Figure 2:**
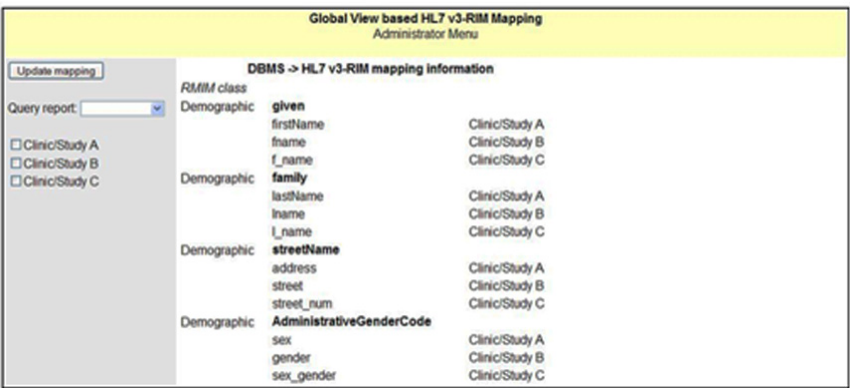
**Mapping information of distributed CDMS/DBMS and RMIM class**. Mapping information of distributed DBMS and HL7 v3 RMIM class.

The key functions of integration workflow are listed:

• Administrator's User Interface (UI) - Administrator interface provides the user an interface to submit queries to the integrated system (HL7 v3-RIM global view).

• SQL optimizer - The submitted queries are used in close collaboration with global view based on HL7 v3-RIM to decompose the query into subqueries which efficiently and effectively follows the stated plan usage to optimize the execution plan for query processing time.

• Global View based HL7 v3-RIM - The R-MIM class represents domain specific healthcare model.

• Relevant Data Extraction - The sub-queries are sent to appropriate distributed legacy CDMS through this function. The partial results are effectively and efficiently combined to finalize the integrated result.

• Web-based Mapping Tool - The online centralized web-based mapping tool allows multiple sites in a clinical trial study to access and perform the mapping between each legacy CDMS and R-MIM classes (See 3.1).

• Legacy to HL7 RIM Mapping Information - The Oracle database engine for data storage and manipulation for legacy CDMS to HL7 RIM mapping information is securely housed in CDMS (Figure 2).

**Figure 3:**
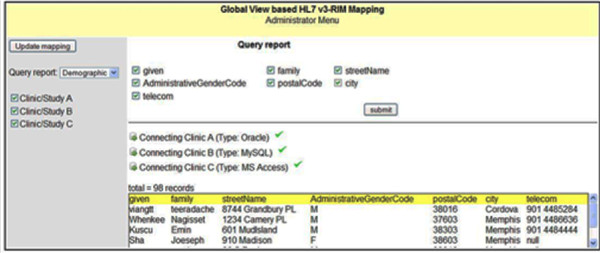
**Web-based Mapping Tool using caAdapter**. Web-based data mapping tool between legacy CDMS and R-MIM using caAdapter.

Once the mapping information is created, the administrator can perform the query against the global view through the UI that has been provided. By consulting through the mapping information, the query is decomposed into subqueries and processed against the global view (HL7 v3-RIM). The partial result extractions are retrieved from each site and combined to form the final result (Figure 3) to be presented to the user with administrative privilege.

### Web-based mapping tool

**Figure 4:**
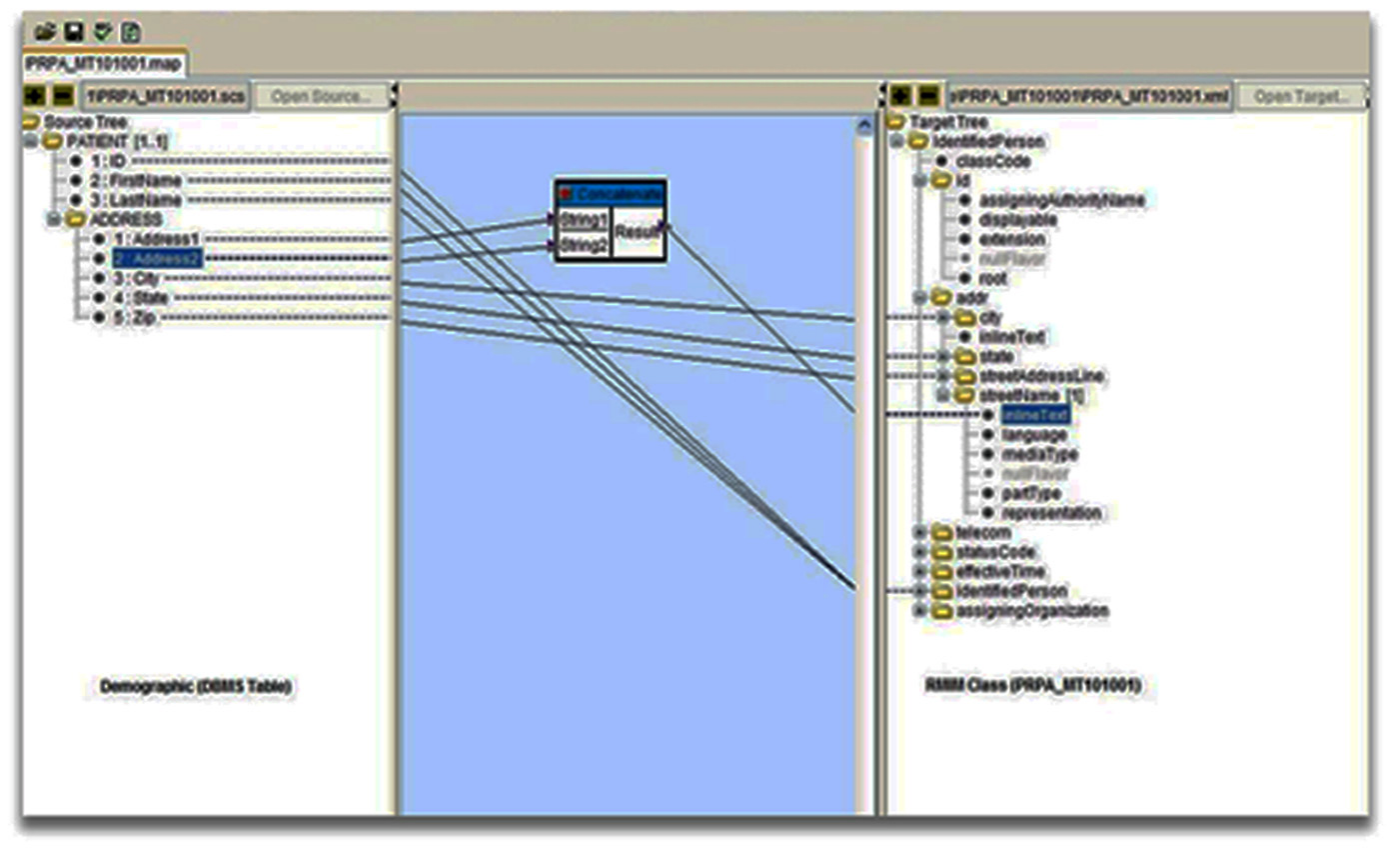
**Web-based Mapping Tool using caAdapter**. Web-based data mapping tool between legacy CDMS and R-MIM using caAdapter.

In the past few years, many groups have devoted a lot of time and effort to develop and design a number of open source, free software and tools for the users who desire to develop and adopt HL7 v3 (RIM). The HL7 Java SIG API (Java SIG group is now the RIM Based Application Architectures (RIMBAA) work group) provides an open source Java Application Program Interface (API) that implements the definition of HL7 v3 RIM [26]. The Open Health Tools (OHT) software repository developed by B2 international (B2i) provides the HL7 v3 Eclipse-based tooling in the package called the Open Health Workbench for v3 message development. It is available for Windows or Mac OS X platforms [27]. The National Cancer Institute Center for Bioinformatics (NCICB) has a set of open source software and tools to support HL7 v3's message generation capability [23]. The NCI/caAdapter HL7 provides the message transformation service (MTS) APIs class written in Java programming language that endeavors to support the users who want to adopt or extend the service into their HL7 v3 message development framework projects. The NCI/caAdapter allows object model-to-model mapping through user interactive and intuitive drag and drop functionality. We decided to adopt caAdapter version 4.3.1 design to support the user with data mapping between legacy CDMS and R-MIM class (Figure 4). The data mapping between legacy CDMS and R-MIM class (Figure 4) can also be explained using the RIMBAA technology matrix [28-32]. It is a 3 by 3 matrix used to describe HL7 v3 application architecture with reference to messaging and other functions of HL7 v3 model like documents exchange and other RIM-based functions. It is also used to show the transition between the cells or squares or the indexing of the message representations which is the XML messages. The 3 by 3 matrix of RIMBAA technology is made up of nine squares or cells with two axes. One of the axes is the Model-View-Controller (MVC) or the model dimension, while the other axis RIM vs. Clone classes vs. Application specific. This axis is used to show the data. The nine cells of RIMBAA are (RP, RO, RS, CP, CO, CS, AP, AO and AS). The cells are defined as follows: AP-Persistent Application specific model, RP - Persistent RIM based mode, CP- Persistent RIM constrained model, RO -Object RIM based mode, CO-Object RIM constrained model, AO-Object Application specific model, RS- Serialized RIM model, CS- Serialized RIM constrained model and AS - Serialized Application specific model.

An HL7 v3 is said to have used RIMBAA technology if at least one of the nine cells is used in message/document transfer or in other RIM-based functions [28-32]. RP, RO and RS cells are called RIM models (generic). CP, CO and CS cells are called RIM constrained information models, while the AP, AO and AS are called application specific models. In this implementation we used CS, CO, and AP squares (Figure 4) to show how the XML messages are being generated and exchanged between compatible systems using the HL7 v3 model.

### HL7 v3 Message Representation

**Figure 5:**
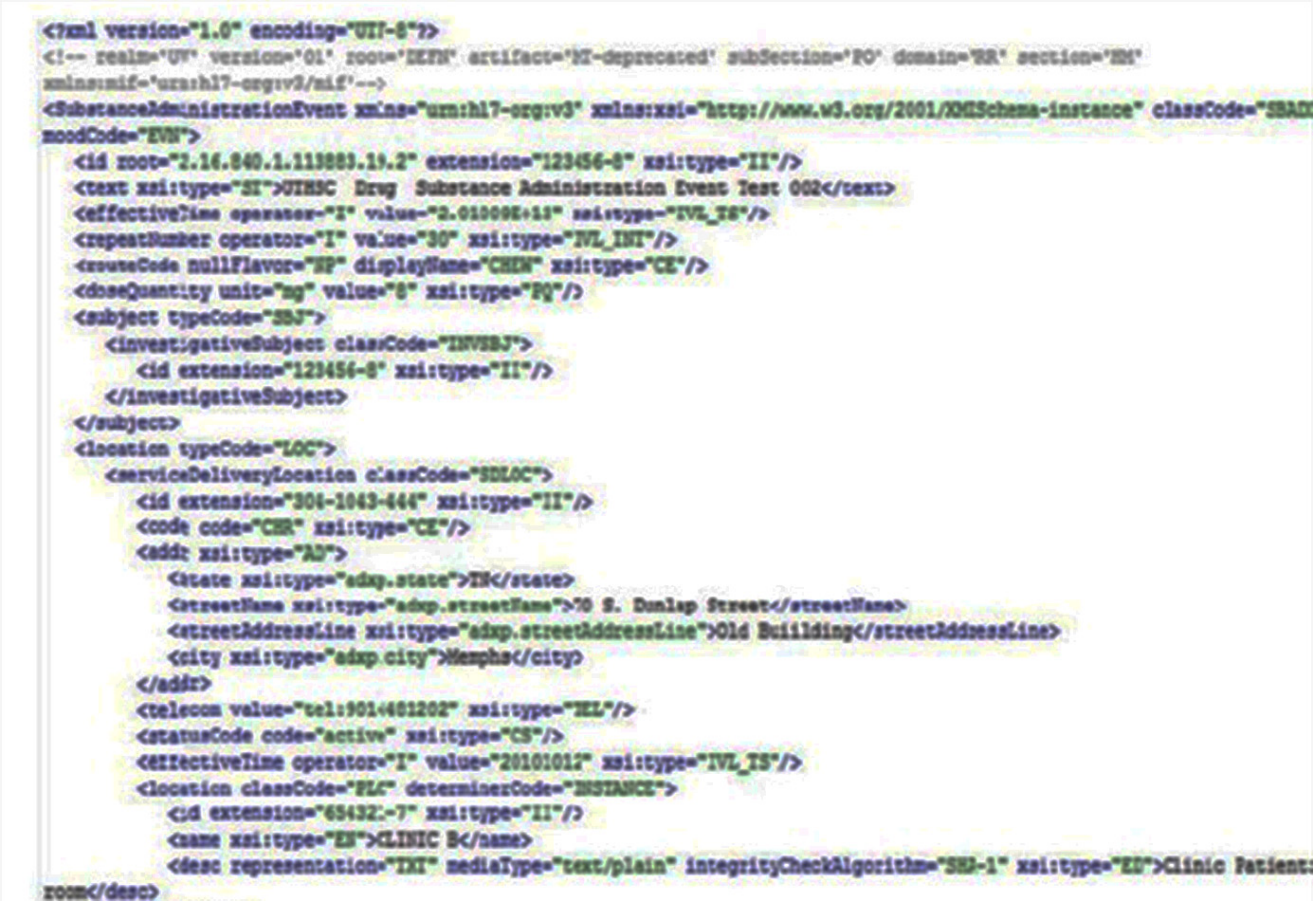
**HL7 v3 message using PORR_MT040002 use case**. Partial result extraction retrieved from each site with HL7 v3 message using PORR_MT040002 use case. **HL7 v3 message using PORR_MT040002 use case (part1)**. Partial result extraction retrieved from each site with HL7 v3 message using PORR_MT040002 use case.

**Figure 6:**
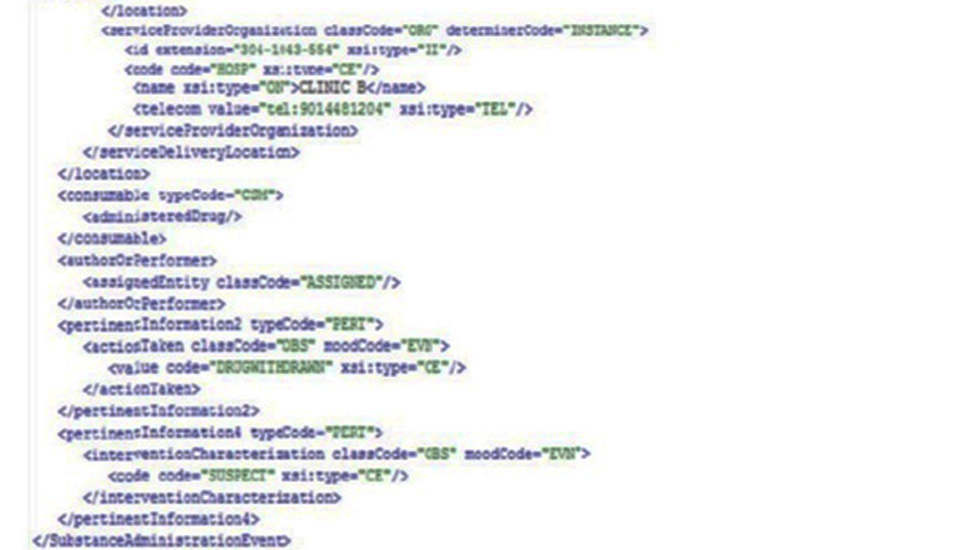
**HL7 v3 message using PORR_MT040002 use case (part2)**. Partial result extraction retrieved from each site with HL7 v3 message using PORR_MT040002 use case.

**Figure 7:**
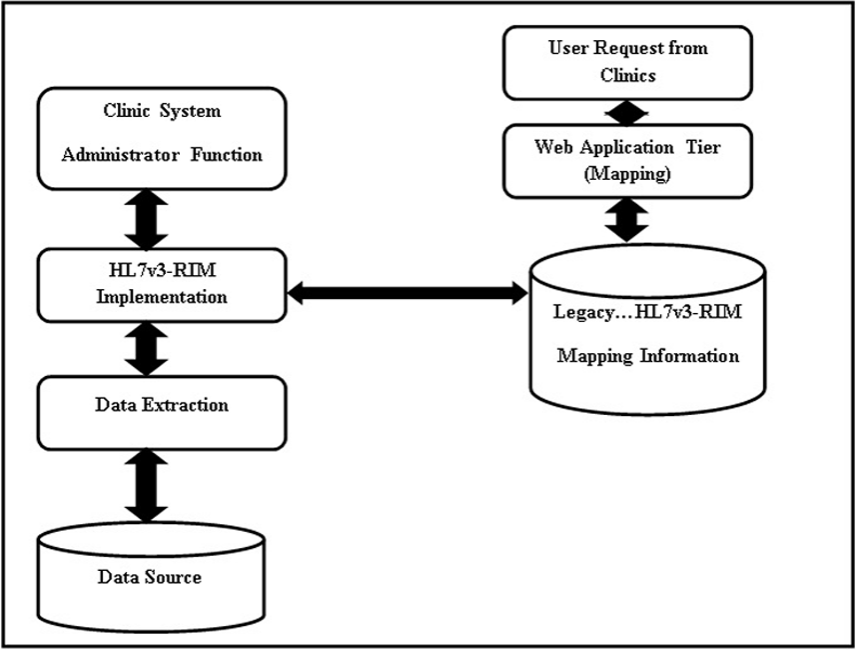
**HL7v3-RIM Implementation Architecture**. The workflow used in the implementation of the prototype.

The HL7 v3 message has been integrated with CDBMS. We implemented a drug administration use case (PORR_MT040002) using clinical trials in three data sources with different underlying database technologies. HL7 v3 messages are generated (Figure 5, Figure 6) in XML format showing the implementation of the drug administration use case for the clinical trials. And the command-line version of HL7 MTS java classes can be downloaded using the link; https://sourceforge.net/projects/hl7mtsdemo/. Figure 7 shows HL7v3-RIM implementation architecture which shows the workflow used in the implementation.

## Discussion

The HL7 v3-RIM development has opened significant movement to support HII and HIE across nationwide healthcare community. However, adopting RIM standard requires a thorough and satisfactory understanding of each and every concept involved. The development of such automation will facilitate mapping between underlying healthcare CDMS and R-MIM and can be used in addressing disparate healthcare system integration issues. The automated mapping service that uses widely accepted methods like pattern matching, semantic mapping and machine learning techniques will enable the mapping process to be dynamic and support real-time collaborative information sharing across translational research community. In addition, this service would minimize the need for manual tracing of all attributes in RIM, which is a difficult and a time-consuming task.

The automated mapping engine is being built on top of the open source Apache Lucene information retrieval library to partially automate the mapping of the legacy database constructs into the relevant D-MIM components. Lucene library provides a rich query language through the query parser that uses JavaCC by Sun Microsystems. Once this process is complete, it will provide term searches, fuzzy searches based on the Levenshtein Distance or Edit Distance algorithm to Proximity [25,33,34]. The dynamic mapping model that is being implemented will replace the Lucene engine to ensure the specificity, relevancy and the security requirements by the clinical database management systems. We are constantly working to improve the designed model to be a software component that can be adopted into various healthcare applications.

Clinical Document Architecture (CDA) is a clinical exchange document authored by HL7. It is based on XML markup structure and used in specifying the standard in terms of syntax, structure and semantics. It is used in creating the HL7 RIM and uses the HL7 v3 data types [23-25]. It is a component of the HL7 version standard and makes use of coding systems like SNOMED, ICD-9, and ICD-10 to represent its concepts. CDA R1 was developed by HL7 in 2000 while CDA R2 was also developed by HL7 in 2005. The difference between CDA R1 and CDA R2 is that, while the former has to do with the structured header, the latter focused on the concepts of the structured elements within the document [35]. It is also used in reading and understanding patient health-care records created by different types of electronic health sources will be used for electronic healthcare systems [12,17]. There are many types of CDA documents created by HL7, e.g., the Continuity of Care Document (CCD), the Cross-Enterprise Sharing of Medical Summary (XDS-MS), the Discharge Summary, etc. A CCD document authored with the American Society for Testing and Materials (ASTM) specifies the constraints of HL7 CDA R2; thus, there is CCR content of the CCD document. The CCR component provides a summary of the patient's clinical, demographic and administrative records [15,19,24,35].

Continuity of Care Record (CCR) is a health information technology exchange standard that is designed, developed and maintained by group of volunteer healthcare information technology professionals under the leadership of ASTM International. It is formally referred to as "E2369-06". CCR contains a digital summary or snapshot of the most relevant of a patient's administrative and clinical health information which can be transmitted or transferred between disparate healthcare systems. It is used in moving medical/health record among facilities (inter-facility movement). It is also based only on XML object oriented relational models [35,36].

HL7 v3 is based on the reference information model (RIM) which has the objective to tackle all the bottleneck involved in the information interchange among health information systems, thus HL7 v3 is used in moving medical/health care records among healthcare facilities (intra-facility movement). The RIM is an abstract model-driven development methodology based on a unified modelling language (UML) and the root of all information models that represent the HL7 data in a standard way across all the domains of healthcare systems. HL7 v3 also uses the XML data model which includes both data and metadata in a unified format. Also HL7 v3 is triggered as a real-time protocol while CCR is not triggered as a real-time protocol [35,36].

Compared with other implementations of health information exchange [9,10,12,13], our prototype implementation of HL7 v3 ensures the mapping for information integration between distributed clinical data sources to promote collaborative healthcare and translational research. This mapping is triggered in a real-time basis to ensure that the right information is received at the right time. Our approach has effectively and efficiently ensured the correctness of the information and knowledge extractions for systems that have been integrated. Our prototype integrates distributed clinical data sources using R-MIM classes from HL7 v3-RIM as a global view, along with a collaborative centralized web-based mapping tool to tackle the evolution of both global and local schemas.

## Conclusion

To assist physicians and other healthcare providers in undertaking and preparing a nationwide health information integration (HII) and health information exchange (HIE) standards, we have made substantial progress, most notably through the creation of a prototype implementation of HL7 v3-RIM mapping for information integration between distributed clinical data sources to promote collaborative healthcare and translational research. Although understanding RIM is necessary for implementing HL7 v3 message standards to solve today's complex healthcare information interchange requirements, the complex standard covers various health information domains extensively and provides a common acceptable standard for HIE. Thus, adopting such standard as a global view will be significant progress in the integration of data from disparate CDMS. We have tested several sample queries against the clinical data integration workflow using the prototype. The approach has effectively and efficiently ensured the correctness of the information and knowledge extractions for systems that have been integrated. In the future we intend to implement the results of the subqueries from the disparate systems that are integrated into a final solution query solution.

## Competing interests

The authors declare that they have no competing interests.

## Authors' contributions

All the authors contributed equally to this work. All authors read and approved the final manuscript
